# Hysterectomy and opportunistic salpingectomy (HOPPSA): study protocol for a register-based randomized controlled trial

**DOI:** 10.1186/s13063-018-3083-8

**Published:** 2019-01-05

**Authors:** Annika Idahl, Anna Darelius, Karin Sundfeldt, Mathias Pålsson, Annika Strandell

**Affiliations:** 1 Department of Clinical Sciences, Obstetrics and Gynecology, Umeå University, Umeå, Sweden; 2 Department of Obstetrics and Gynecology, Institute of Clinical Sciences, Sahlgrenska Academy at University of Gothenburg, Gothenburg, Sweden

**Keywords:** Ovarian cancer, Opportunistic salpingectomy, Early menopause, Hysterectomy, Complications, Menopausal symptoms

## Abstract

**Background:**

There is a great need for a prospective randomized trial to evaluate the risks and benefits of opportunistic salpingectomy. Recently, genetic and morphologic studies have indicated that epithelial ovarian cancer predominantly develops in the Fallopian tubes. Consequently, there is reason to believe that salpingectomy would reduce the risk of ovarian cancer. Studies on reducing the risk of ovarian cancer have compared indicated salpingectomy with no salpingectomy, while studies on surgical safety as well as ovarian function after opportunistic salpingectomy have been small with a short follow-up. No study has reported menopausal symptoms.

**Methods/design:**

In this national register-based randomized controlled trial, women <55 years old, planned for a hysterectomy for a benign cause, will be randomized to concomitant salpingectomy or no salpingectomy. The follow-up will be conducted according to already established routines within the register using on-line questionnaires. Primary outcomes have been defined for three different time points: short-term complications up to 8 weeks postoperatively (*n* = 2800), intermediate-term changes in menopausal symptoms measured by the Menopause Rating Scale at baseline and after 1 year (*n* = 1670), and long-term epithelial ovarian cancer assessed through national registers after 30 years (*n* = 5052) (or *n* = 7001 for high-grade serous cancer). In a sub-study of 75 women, ovarian function will be evaluated through change in anti-Müllerian hormone measured before surgery and after 1 year.

**Discussion:**

Hysterectomy for a benign cause is a common surgical procedure and several national societies recommend salpingectomy while performing a benign hysterectomy, despite a lack of scientific evidence for the safety of the procedure. Sweden has unique conditions for clinical trials because of its national quality registers and health registers with excellent quality and near complete coverage. If no additional risks are associated with concomitant salpingectomy, it can be recommended at the time of benign hysterectomy to reduce the risk of epithelial ovarian cancer. If not, the risks and benefits must be balanced. The results of this study will be important for informing women undergoing a benign hysterectomy.

**Trial registration:**

ClinicalTrials.gov, NCT03045965. Registered on 8 February 2017.

## Background

Performing an opportunistic salpingectomy during a benign hysterectomy in the general population to reduce the risk of epithelial ovarian cancer (EOC) has not yet been thoroughly evaluated. Evidence is lacking not only on the actual reduction in the number of EOC cases, but also on the feasibility, safety, and possible negative effects on ovarian endocrine function of the procedure. A large prospective randomized controlled trial is needed to estimate both short- and long-term effects, such as surgical complications, endocrine function, and EOC risk reduction.

New ways to decrease the mortality of EOC, which is a gynecological malignancy, are urgently required. Approximately 240,000 new cases are diagnosed globally each year, and 152,000 women die each year from the disease [1]. In Sweden, 544 new cases and 556 deaths were registered during 2016 [2]. The disease is mostly diagnosed at a late stage and consequently has a poor prognosis. Despite progress in surgical techniques and chemotherapy, we need to explore new approaches for prevention, screening, and early diagnosis.

In the general population, oral contraceptive use is associated with a 40–50% lifetime risk reduction of EOC [3, 4]. Multiparity and long lactation periods have also been considered protective against EOC [5, 6]. An increased risk of EOC is associated with inflammatory diseases like salpingitis [7, 8] and endometriosis [9]. Furthermore, chronic inflammation, pelvic inflammatory disease, and endometriosis contribute to the progression of ovarian cancer [10]. Bilateral salpingo-oophorectomy is a preventive strategy for reducing the risk of EOC in women with a family history of ovarian cancer (BRCA1 or BRCA2 mutation carriers) after childbearing is completed. This procedure dramatically decreases the incidence of EOC [11]. However, an oophorectomy in premenopausal women will induce the menopause, which confers an increased risk of cardiovascular morbidity, osteoporosis (fractures), and symptoms of reduced estradiol (hot flushes and changes in sexual function). Thus, for premenopausal women without a family history of ovarian cancer, the benefits of an opportunistic oophorectomy at the time of hysterectomy for a benign indication do not outweigh the risks [12], and therefore, this procedure is not recommended.

Our understanding of the etiology of EOC has changed during the last 15 years and high-grade serous cancer (HGSC) is today considered to originate in the Fallopian tube (see the review by Kurman and Shih [13]). EOC includes a group of extremely heterogeneous carcinomas, all with differences in origin, molecular biology, morphology, gene expression, and behavior. EOC is at least five distinct diseases (HGSC, low-grade serous cancer, endometrioid cancer, clear-cell cancer, and mucinous cancer), all with differences in morphology, molecular biology, intrinsic gene expression, and the diversity of biologic behavior [14]. For HGSC, the most common histology, the Fallopian tubes have a central role, and the ovary is seldom the origin but is instead involved secondarily. HGSC produces no defined preclinical lesions in the ovaries. However, precursor lesions called serous tubal intra-epithelial carcinoma, and an early alteration in TP53 gene function before the serous tubal intra-epithelial carcinoma, have been found in the epithelium of the distal fimbria of the Fallopian tube [15], which were first detected in patients with the BRCA1 or BRCA2 mutations [16]. The serous tubal intra-epithelial carcinoma lesions from the tubal fimbria are assumed to implant onto ovarian or peritoneal surfaces, and after an occult period, develop into a fast-growing HGSC. Immunohistochemical, morphologic, and molecular genetic analyses indicate that these lesions are metastases [15, 17–19]. It has also been suggested, but not widely accepted, that endometrioid and clear-cell carcinomas develop from the endometrium and endometriosis due to retrograde menstruation and they also involve the ovary secondarily [19–21]. Based on these data, a salpingectomy could potentially reduce the risk of EOC substantially.

Two retrospective observational epidemiological studies in Sweden and Denmark have suggested that there was a relative reduction in the risk of ovarian cancer after salpingectomy due to pathological tubes compared to no surgery of 35–42% [22, 23]. Concomitant bilateral salpingectomy at the time of benign surgery, such as a hysterectomy or tubal ligation, has, thus, been strongly recommended for the general population in some countries. Even though this is appealing, the data to substantiate this procedure are missing. It might seem unharmful to remove the Fallopian tubes after childbearing is completed but difficulties with the surgical procedure for removing all fimbriae have been reported [24]. In a small randomized study of 100 women looking at anti-Müllerian hormone (AMH) levels postoperatively as the primary outcome, 10% experienced failure of the intervention and complications were 50% higher in the intervention group than in the control group [25]. One observational study found an increased surgical time (16 min) but no increase in the need for blood transfusions or hospital readmissions [26]. Abdominal as well as laparoscopic and vaginal surgical approaches were evaluated. Neither Vorwergk and colleagues nor Morelli and colleagues found any increase in surgical complications in their retrospective cohort studies [27, 28]. These findings strongly urge us to continue with our large prospective register-based randomized controlled trial.

The International Federation of Obstetrics and Gynecology (FIGO) consists of 130 member societies. In a recent survey of member societies from 2018 on opportunistic salpingectomy, only 14 countries had guidelines. Nine of these support consideration of opportunistic salpingectomy in appropriate women and four (Sweden, Norway, France and, Germany) are ambivalent [29]. Based on a systematic review from 2016 initiated by the Swedish Society of Obstetrics and Gynecology, we concluded that data were lacking on both the advantages and the disadvantages of the procedure, even though research does not question the pathological role of Fallopian tube precursor lesions in tumorigenesis [30].

This study protocol aims to examine hysterectomy and opportunistic salpingectomy (HOPPSA) using a register-based randomized controlled trial. The study has a non-inferiority design for the primary endpoints of complications after 8 weeks and menopausal symptoms after 1 year. Epithelial ovarian cancer risk reduction will be studied in a superiority design as a long-term primary endpoint.

## Methods/design

### Aims

The aims of the present trial are to examine, in a national register-based randomized controlled trial, whether opportunistic salpingectomy compared with no salpingectomy, at the time of a hysterectomy for a benign reason

- has no increased risk of complications
- has no negative side effects on ovarian function that may result in an earlier menopause
- implies reduced risk of subsequent EOC.

### General design

The study is a national register-based randomized controlled trial and conducted within the Swedish National Quality Register of Gynecological Surgery (GynOp) [31], which has data from the vast majority (>95%) of gynecological departments in Sweden. All gynecological departments reporting data to the registry have received written and oral information about the trial and will automatically be included in the study unless the clinic has reported their wish to abstain. Inclusion and participation in national quality registers in Sweden is clearly regulated by law [32], which stipulates that all patients are to be included in the register. However, a patient can decline (opt out). The GynOp database is approved for use by health-care systems under the supervision of the Swedish Data Protection Authority and if they hold the highest certification level. Background health data and information on surgical procedures, diagnoses, and complications up to 1 year postoperatively are routinely recorded in GynOp. The data collection forms and questionnaires are available from www.gynop.org on request. Information, questions, consent, and group allocation specific to this study have been added to the GynOp application. A list of participating clinics can be provided by the GynOp office in Umeå if contact is made through the website.

The study has a non-inferiority design for the short- and intermediate-term primary outcomes. A superiority design is applicable for the long-term primary outcome *time to ovarian cancer*. All three primary objectives (to show non-inferiority of *complications* at 8 weeks and non-inferiority of *absolute change in menopausal symptoms* after 1 year, as well as superiority of *time to EOC* after up to 30 years) must be fulfilled to prove the study hypothesis.

**Fig. 1:**
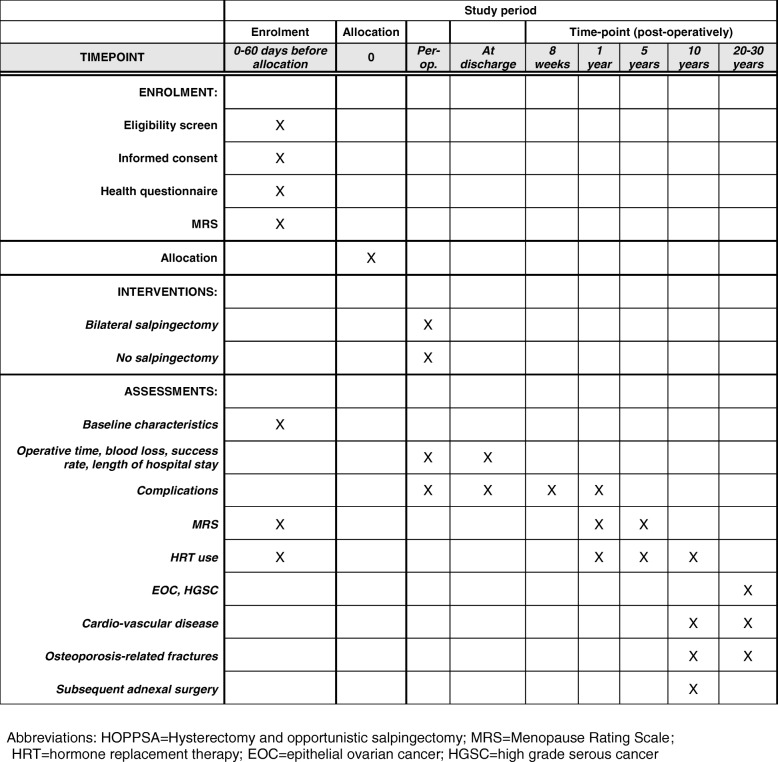
Schedule of enrolment, interventions, and assessments in the HOPPSA trial according to the SPIRIT guidelines

A SPIRIT figure and checklist for this study protocol are provided in Fig. 1 and Additional file 1, respectively.

### Participants

All patients planned for hysterectomy due to a benign indication will be automatically screened for eligibility within GynOp after the planned surgical procedure has been registered. Women must be younger than 55 years at the time of randomization. Exclusion criteria are previous bilateral oophorectomy or salpingectomy and planned oophorectomy or salpingectomy (for reasons such as an already diagnosed adnexal tumor, known carrier of the BRCA1 or BRCA2 mutation, or Lynch syndrome). Women who do not understand the oral or written study information will not be included.

Patients routinely receive a personal password that allows them to log on to GynOp to answer the health questionnaire. At the same time under the protection of the password, they can read the HOPPSA study information, answer the specific study questions, and give or refuse consent for participation in the study. The patients may also complete a paper version of this form, which will subsequently be entered into GynOp by a secretary using a logon with SITHS identification (a two-factor authentication system for safe identification).

### Randomization

The randomization to the two groups (salpingectomy or no salpingectomy) will be performed within GynOp using a dedicated randomization module. The randomization will be performed in a 1:1 ratio. The randomization will be stratified for the variables: center, age (<50 and ≥50 years), and intended operative route (abdominal, laparoscopic, or vaginal). The vaginal route will be included only for those clinics that already perform bilateral salpingectomy vaginally. The randomization program will ensure that an even distribution between randomization groups occurs over time by applying variable block sizes. The timing of randomization will be as close as possible to the time of surgery, i.e., at the preoperative visit, closest to the day of surgery, or on the day of surgery, depending on local practical circumstances. Concealed allocation is guaranteed by the randomization program. If the inclusion criteria have been fulfilled and informed consent has been received, the examining/operating gynecologist or an assistant who has logged on to GynOp can perform the randomization. The result is immediately presented on the screen.

### Intervention

Bilateral salpingectomy will be performed in conjunction with hysterectomy according to clinical practice. If randomized to no salpingectomy, the hysterectomy will be performed leaving the adnexa in place. If a previously unknown adnexal pathology is discovered perioperatively, the appropriate surgery will be conducted and registered in GynOp, without excluding the patient from the follow-up.

### Blinding

The initial intention was to blind patients to the surgical procedure until 1 year after surgery (after the evaluation of post-menopausal symptoms). However, blinding is not possible since patients will be able to read their medical records on-line. The benefit of blinding will be explained to patients. Patients will receive information on the surgical procedure performed if they so wish.

### Outcomes

There are three primary endpoints, each with a different time perspective. Complications related to surgery in the short-term perspective will cover the period up to 8 weeks after surgery. Data will be retrieved from the routine questionnaire in GynOp, including the Clavien-Dindo classification [33] and specific questions on complications answered by the patients and assessed by the doctor.

As intermediate-term outcome, the absolute change in menopausal symptom score, measured from the baseline to the 1-year follow-up, will be assessed with the Menopause Rating Scale (MRS) [34] in GynOp. MRS is a validated questionnaire available in several languages, including Swedish. It has 11 questions on sweating, heart discomfort, sleep problems, depressive mood, irritability, anxiety, physical and mental exhaustion, sexual problems, bladder problems, vaginal dryness, and joint and muscular function, to which patients respond in a five-grade scale [35].

The long-term outcome, time to EOC (specifically HGSC) including primary tubal and peritoneal cancer, will be assessed through the Swedish Cancer Register, the Swedish Quality Register for Gynecological Cancer, the Swedish Cause of Death Register, and the Swedish Population Register, up to 30 years after surgery.

Secondary short-term outcomes are measured and registered in GynOp in connection with the hospital stay: operative time, length of hospital stay, perioperative blood loss, conversion to other surgical route, and failure rate of salpingectomy during planned vaginal hysterectomy. Secondary intermediate- and long-term outcomes include complications after 1 year, menopausal symptoms in absolute and relative measures at 1 and 5 years, subsequent adnexal surgery and use of hormone replacement therapy within a 10-year period, and cardiovascular events and osteoporosis-related fractures from 10 to 30 years. These will be analyzed using data from GynOp and other national registers (the Patient Register and the Prescription of Drug Register).

### Follow-up and monitoring

Variables will be registered continuously according to present routines in GynOp. The routines include assessments preoperatively, at discharge from hospital, and at 8 weeks and 1 year postoperatively. In an assessment, the patient replies to questions and her doctor has the responsibility for arranging any clinical follow-up, if needed. Any complication or complaint assessed at a clinical visit will be documented in GynOp. A 5-year follow-up questionnaire will be added to GynOp for the study. If the postoperative course is uneventful, the follow-up will be conducted entirely through the GynOp questionnaires.

Response rates in GynOp for 2012–2015 were 82% at 8 weeks and 78% at 1 year. Two reminders are sent routinely. Study participants will receive additional reminders by mail, e-mail, and phone to reduce the attrition rate. Efforts to ensure we have complete data for each patient for the primary outcomes will be accomplished firstly at the local hospital level by doctors who have a local responsibility for HOPPSA and who are the local representative of the Swedish Network for National Clinical Studies Within Obstetrics and Gynecology (SNAKS), and secondly by the steering group. The SNAKS representatives and the local HOPPSA gynecologists will check responses and the completeness of the questionnaires at the different time points (8 weeks, and 1 and 5 years).

The number of women randomized will be continually monitored at the GynOp office in Umeå and this will govern the duration of the study. Long-term data will be retrieved from the relevant registries according to the planned follow-up.

### Sample size calculations

Approximately 2800 women < 55 years of age who underwent abdominal or laparoscopic hysterectomy for a benign reason were registered in GynOp during 2016. This is a non-inferiority design with sequential analyses of the short- and the intermediate-term primary outcomes— *complications* at 8 weeks postoperatively and *absolute change in menopausal symptoms* 1 year after surgery. A superiority design is planned for the long-term outcome of incidence of EOC, particularly HGSC.

### Short-term outcome: *complications* at 8 weeks (non-inferiority)

The overall complication rate (mild and severe) was 30% during 2015. If non-inferiority is defined as an increase in the complication rate of up to 8%, the upper limit of the two-sided 95% confidence interval (CI) for the difference between groups will not exceed 8% with a probability of 80% (β = 20%). With an estimate of up to 3% more complications in the salpingectomy group, 1280 patients per randomization group are needed to show non-inferiority.

A small proportion of patients will not undergo the allocated procedure (estimated to be at most 3%), due to unsuspected tubal or ovarian pathology and difficulties in performing the intended salpingectomy. Moreover, based on data from GynOp for 2012–2015, the attrition rate at 8 weeks was 18% (82% response rate from patients). We expect a much lower loss to follow-up within the study (estimated at 4%), since the HOPPSA representative at each clinic will be responsible for completing the data in GynOp. Thus, the total expected loss to follow-up is estimated to be a maximum of 3 + 4 = 7%. To allow for a 7% loss to follow-up, we need to recruit approximately 2800 patients to give 2560 patients evaluable for analysis (1280 per group). The sample size target will include the laparoscopic and abdominal routes. The vaginal route is likely to have a high failure rate and will not be included in the target sample size.

If all clinics that register in GynOp participate and 80% of eligible patients consent to randomization (*n* = 2240 per year), recruitment will require 1.3 years. Correspondingly, if 50% consent (1400 patients per year), recruitment will take 2 years.

### Intermediate-term outcome: *absolute change in menopausal symptoms* at 1 year (non-inferiority)

MRS gives a rating of 0–4 for 11 items, resulting in a total range of 0–44. An increase in MRS from baseline to 1 year is expected in both groups, based on the negative effect of a hysterectomy on ovarian function [36] and also due to the participants’ increasing age. A clinically relevant difference in MRS is defined as 5 points [37]. This difference can be applied both as a clinically relevant change within groups as well as a clinically relevant difference in change between groups. If non-inferiority is defined as 4 points, the upper limit of the two-sided 95% CI for the difference in change between the two groups will not exceed 4 points (standard deviation for change 6.9) with a probability of 80% (β = 20%). With an estimate of up to 3 additional points in the salpingectomy group, 749 patients per randomization group are needed to show non-inferiority.

Based on data from GynOp for 2012–2015, the attrition rate at 1 year was 22% (78% response rate from patients). We expect a much lower loss to follow-up within the study (estimated at 7%), since several more attempts than the routine reminders to patients will be performed. Also, taking into account the estimated rate of protocol violations of 3% during surgery, as described above, the total attrition rate for this outcome is estimated to be at most 3 + 7 = 10%. To allow for a 10% loss to follow-up, we need to recruit approximately 1670 patients, to give 1500 patients evaluable for analysis (750 per group). Thus, the sample size for the outcome *complications* greatly exceeds the sample size needed for the outcome *absolute change in MRS*.

### Long-term outcome: *time to EOC* (superiority)

The number of new EOC cases in Sweden is approximately 630 per year, of which approximately 440 are the fatal subtype HGSC. The overall lifetime risk of being diagnosed with ovarian cancer is approximately 2%, with EOC is 1.8%, and with HGSC is 1.3%. If the incidence is reduced by 50% to 0.9% for EOC (0.65% for HGSC), given β = 20%, α = 5%, and a two-sided test, we need 2412 patients per group to demonstrate superiority for EOC (3343 for HGSC). The calculation is based on survival analysis (log-rank test) with 4 years of accrual time and 30 years of follow-up. The 30-year survival rates for EOC are assumed to be 98.2% (no salpingectomy) and 99.1% (bilateral salpingectomy) in the two randomization groups respectively (98.7% and 99.35% for HGSC) and we assume an increasing hazard rate. The estimated risk of loss to follow-up is low since the outcome data will be from the National Board of Health and Welfare. Assuming a 1.5% loss to follow-up and 3% excluded due to non-eligibility detected after randomization (at surgery), 5052 patients will be needed for the EOC outcome (7001 for the HGSC outcome). Thus, the total sample size of 5052 for EOC and 7001 for HGSC for the long-term outcome *ovarian cancer* exceeds the sample size for the primary outcome *complications* (2800).

### Overall recruitment

To allow for an adequate analysis of the randomized cohort for EOC and HGSC, the number of participants recruited needs to be extended from that required for the short-term outcome (2800) by approximately 2250 for the EOC outcome and 4300 for the HGSC outcome, corresponding to an approximately 1–1.6 extra years of recruitment for EOC and 2–3.2 extra years for HGSC. The independent Data and Safety Monitoring Board will decide whether it is feasible to continue recruitment to reach the target sample size of 7001 patients or to stop at 5052, based on the recruitment rate and the available results for the short- and intermediate-term safety outcomes.

### Statistical methods

For descriptive statistics, the mean, standard deviation, median, and first and third quartiles will be used. Protocol violations are expected for patients randomized to no salpingectomy when unsuspected tubal or ovarian pathology is apparent at surgery and for patients randomized to a salpingectomy that was not performed due to surgical difficulties. All patients will be followed according to the protocol. Both intention-to-treat and per protocol analyses will be performed. For the non-inferiority design, the primary analysis will be per protocol. For the superiority design, the intention-to-treat analysis will be the primary analysis. Patients with unsuspected tubal or ovarian pathology detected at surgery that is indicative of a salpingectomy or an oophorectomy will be excluded from the analysis of the prespecified subgroup of patients eligible for a true opportunistic salpingectomy.

Baseline characteristics will be compared between responders and non-responders to evaluate the risk of attrition bias. The main analysis will be with multiple imputations for missing data. Sensitivity analyses will be conducted, using complete cases without imputation (full analysis dataset).

In the comparison between the two groups, we will use Student’s *t*-test for baseline continuous variables, the Mantel–Haenszel chi-squared test for ordered categorical variables, and Fisher’s exact test for proportions for both baseline and outcome variables. Tests for superiority will be two-sided and performed at a significance level of 0.05. For comparisons within the randomized groups, we will use the Wilcoxon signed-rank test for continuous variables and the sign test for ordered or dichotomous variables.

The secondary outcomes will be analyzed descriptively and presented with unadjusted *p* values. None of the secondary outcomes will be confirmatory.

### Main analyses

For the outcome *complications* (non-inferiority design), a two-sided 95% CI for the difference in proportions of complications between randomization groups will be constructed according to the method of Miettinen and Nurminen [38]. The lower limit of the 95% CI will not exceed the non-inferiority margin of 8%. Missing data will be handled using fully conditional multiple imputation in the main analysis. A sensitivity analysis will be performed with the full analysis dataset without imputation.

For the outcome *absolute change in MRS score* (non-inferiority design), a two-sided 95% CI for the mean difference in change in MRS will be constructed using analysis of covariance (ANCOVA) with baseline MRS score and age as covariates. The lower limit of the 95% CI will not exceed the non-inferiority margin of 4 points. Missing data will be handled using fully conditional multiple imputation in the main analysis. A sensitivity analysis will be performed with the full analysis dataset without imputation.

The outcome *time to epithelial ovarian cancer* (superiority design) will be compared between the two randomization groups using the log-rank test at a significance level of 0.05.

### Complementary analyses

The three primary outcomes will undergo complementary analyses with adjustment for prespecified baseline variables, which will be defined in the final statistical analysis plan. A generalized *estimating equation* analysis with a log link function will be used for dichotomous outcomes and ANCOVA will be applied for continuous outcomes. Cox regression models will be used to calculate the hazard ratio with 95% CI.

### Exploratory prediction analyses

Prediction models will be built for all three primary outcomes as an exploratory aim. They will not be included in the analyses of the randomized results. For the outcomes *complications* and *change in MRS score*, since they are dichotomized variables, the models will be chosen according to the Akaike information criterion and the results will be presented as odds ratio with 95% CI. Cox proportional regression models will be used to find independent predictors of the development of EOC. The model with the lowest Akaike information criterion will be selected. The model building will include cross-validation and a minor part of the study population will be used for external validation of the model.

#### Sub-study of AMH levels

To strengthen the hypothesis of non-inferiority for ovarian function if salpingectomy is performed, an analysis of serum AMH is planned in a nested trial within HOPPSA. Blood samples will be taken at the baseline and after 1 year. The samples will be frozen and stored in a biobank for later analysis. The entire cohort will be analyzed at the same time.

The primary outcome is *absolute change in AMH*. Secondary outcomes are *relative change in AMH* and *level of AMH* 1 year after surgery. If non-inferiority is defined as 0.125 mg/L AMH, the higher limit of the two-sided 95% CI for the difference in change between the two groups will not exceed 0.125 (standard deviation for change 0.1) with a probability of 80% (β = 20%). With an estimate of AMH levels being up to 0.05 larger in the salpingectomy group, we need 29 patients per randomization group to show non-inferiority. Estimating a 20% loss to follow-up (a second blood sample not taken), 74 patients will be recruited into this nested trial. A two-sided 95% CI for the mean difference in *absolute change in AMH* will be constructed using Fisher’s exact permutation test.

## Discussion

The HOPPSA protocol is for a register-based randomized controlled trial utilizing a national quality register, GynOp, for inclusion, randomization, and follow-up. The underlying research question is whether opportunistic salpingectomy prevents future epithelial ovarian cancer. The questions that will be tested first are whether the intervention is harmful due to complications related to surgery or changes in ovarian function. Thus, the trial has three primary outcomes covering the short, intermediate, and long terms. The choice of primary outcomes is based on whether the potential reduction in the risk of EOC can be obtained by opportunistic salpingectomy without negative consequences in the short- and intermediate-term outcomes.

Ovarian cancer is a heavy burden for the affected individual, but also for society, since treatment is costly and often has an unfavorable outcome. Obviously, the possibility of reducing the risk of developing a very serious and often fatal condition must be of high importance. If such risk reduction is possible without simultaneously increasing the risk of complications or premature menopause, much is gained for the individual woman. A potential reduction in the incidence of EOC due to opportunistic salpingectomy is, therefore, highly desirable.

A long time will be required to address the long-term primary objective. The aim is to recruit a large enough randomized cohort that we can detect a reduction in the incidence of HGSC. The Data and Safety Monitoring Board will make the decision on whether to stop or continue recruitment based on safety. If recruitment is stopped before the optimal sample size is reached for the primary outcome HGSC, the target number will be reduced so that the sample size is sufficient for analyzing EOC and also overall ovarian cancer.

However, results from the short-term outcome (complications) and the intermediate-term outcome (change in menopausal symptoms) will be available within a few years. If non-inferiority is demonstrated, opportunistic salpingectomy can be recommended, despite that the results for EOC are not available. On the other hand, if non-inferiority is not demonstrated, i.e. if either surgical or hormonal complications are increased after salpingectomy, women planned for hysterectomy need to be fully informed of the increased risks with the procedure, allowing them to make an informed decision concerning concomitant salpingectomy.

There are several advantages of using this register-based study design. GynOp has an almost 100% national coverage. Virtually all clinics performing hysterectomies in Sweden already use the register. Also, patients usually answer the follow-up questionnaires. The GynOp platform has a high level of automatization. Questionnaires are made available to the patients electronically, but there is also a well-established routine to offer questionnaires in paper format when requested.

A specific trial module has been built into the GynOp register. The module issues a notification when a potentially eligible patient is registered in GynOp, provides the patient with study information, and facilitates randomization, including stratification and the concealed allocation. The ability to add functionality for this study into the already established platform GynOp was, therefore, central to the design of the study.

Sweden as a country is uniquely well suited for this type of study, since all of its citizens are given a personal identification number at birth or immigration. This enables easy and reliable linkage between different registers. The HOPPSA trial collects data from GynOp, the Patient Registry, the Swedish Cancer Registry, the Swedish Quality Register of Gynecological Cancer, and the Prescribed Drug Registry. In Sweden, the authorities have stipulated that all patients should be included in quality registers (unless they actively decline) to monitor the quality of care provided, but also to create databases for research. This means that most women planned for benign hysterectomy in Sweden will be reached and screened for eligibility.

The follow-up is conducted completely within the registers, without any need for checking medical records or personally contacting patients, a feature of great importance for the expected low attrition rate in the long term. Furthermore, the HOPPSA trial is supported by SNAKS. All gynecological departments in Sweden are members of the SNAKS network, enabling fast and comprehensive communication to all participating clinics.

The main challenges of the HOPPSA trial are the multi-center design and that some clinicians already undertake an opportunistic salpingectomy when performing a hysterectomy. Being multi-center is a strength and also a weakness. It is challenging to keep all participants up to date and for the centers to be active in recruiting patients. The GynOp register will be an important help in maintaining awareness of the trial, since study information is given to eligible patients automatically when they receive the preoperative questionnaire. Moreover, the follow-up uses the already established GynOp logistics. Additionally, information and support provided to local representatives from the research group and through the SNAKS network will be crucial for maintaining a high level of patient recruitment.

Some countries have already issued recommendations to perform opportunistic salpingectomy when performing a benign hysterectomy. In Sweden, no such national guidelines have been issued due to the knowledge gap on the safety of opportunistic salpingectomy. If the HOPPSA trial can show that there are no differences in the short- and intermediate-term outcomes, national guidelines for opportunistic salpingectomy at the time for benign hysterectomy can be issued. If the study indeed shows a difference in the outcomes, favoring a conservative approach, the findings can be used to give women undergoing a hysterectomy information based on sound science when making their informed decision.

### Trial status

The final version of the protocol is dated 25 August 2017. This version includes an amendment that specifies that *EOC* is the appropriate type of ovarian cancer to be used in the sample size calculation. This amendment was approved by the regional ethical review board in Gothenburg. The first patient was randomized on 14 June 2017. Preliminary calculations estimate recruitment to be completed 31 December 2022, or earlier if appropriate according to the DSMB.

## Additional file

Additional file 1: Items addressed in HOPPSA - Hysterectomy and opportunistic salpingectomy according to the SPIRIT 2013 Checklist. (DOC 120 kb)

## Abbreviations

AMH: Anti-Müllerian hormone
ANCOVA: Analysis of covariance
CI: Confidence interval
EOC: Epithelial ovarian cancer
FIGO: International Federation of Obstetrics and Gynecology
GynOp: Swedish National Quality Register of Gynecological Surgery
HGSC: High-grade serous cancer
HOPPSA: Hysterectomy and Opportunistic Salpingectomy
MRS: Menopause Rating Scale
SNAKS: Swedish Network for National Clinical Studies Within Obstetrics and Gynecology
